# The Associations of Body Mass Index, Body Image, Perceived Stress, and Mental Health among Female Nursing Students: A Cross-Sectional Study in Taiwan

**DOI:** 10.3390/healthcare11172426

**Published:** 2023-08-30

**Authors:** Ching-Feng Huang, Fan-Hao Chou, Chia-Hao Chang, Su-Er Guo

**Affiliations:** 1 Department of Nursing, Chiayi Campus, Chang Gung University of Science and Technology, Chiayi County 613016, Taiwan; cfhuang@mail.cgust.edu.tw (C.-F.H.); chchang@mail.cgust.edu.tw (C.-H.C.); 2 College of Nursing, Kaohsiung Medical University, Kaohsiung City 807378, Taiwan; 3 Department of Nursing, Chronic Diseases and Health Promotion Research Center, Chiayi Campus, Chang Gung University of Science and Technology, Chiayi County 613016, Taiwan; seguo@mail.cgust.edu.tw

**Keywords:** body mass index, body image, perceived stress, mental health, nursing students

## Abstract

Maintaining a healthy caloric intake and expenditure balance is challenging. The preliminary study examined (a) the associations of Body Mass Index (BMI), body image, perceived stress, mental health, dietary habits, and exercise participation among 310 female college nursing students and (b) the differences in these factors among BMI subgroups. The cutoffs for underweight, normal weight, overweight, and obesity were <18.5 kg/m^2^, ≥18.5 but <23 kg/m^2^, ≥23 but <25 kg/m^2^, and ≥25 kg/m^2^, respectively. The survey used Multidimensional Body-Self Relations Questionnaire—Appearance Scale, Perceived Stress Scale, and Chinese Health Questionnaire (CHQ). Because of a non-normal distribution, non-parametric statistics were used. The study found that BMI correlated with body image. The lower the BMI, the higher the satisfactory score the participants rated. BMI was irrelated to diet, exercise, stress, and mental health. The participants were unlikely to eat under stress and negative moods. The underweight group (17.1%) had the highest satisfactory score on their appearance evaluation. There was no difference between the overweight (14.2%) and obese (11.0%) groups. Meanwhile, the median of perceived stress was 18 and the prevalence of mental disturbance was 45.2% based on the CHQ. Underweight, overweight, obesity, high perceived stress, and poor mental health among nursing students warrant attention. Nurse educators should strengthen their coping strategies and provide support. A longitudinal study may consider incorporating coping strategies into the study design.

## 1. Introduction

Overweight and obesity caused 2.7–5.3 million deaths worldwide in 2015 [1]. Among the deaths, approximately two-fifths occurred when the people were not obese [1]. Overweight and obesity increase the risks of cardiovascular and musculoskeletal diseases, diabetes, and cancers [2]. The global rates of overweight and obesity were approximately 39% and 13% among people aged ≥18 years in 2016 [2].

Diet and exercise are fundamental factors causing disequilibrium between caloric intake and expenditure. Personal preferences and environmental factors may influence dietary habits. People may prefer food and drinks with high fat and carbohydrate/sugar levels, resulting in excessive caloric intake and increased body weight [3,4]. Although take-out services and campus-based cafeterias usually provide food and drinks with high fat and carbohydrate/sugar levels, people may choose them because of their easy access and time-saving nature [3,4].

Meanwhile, female college students are more likely to be underweight [3,5], which increases the risk of sarcopenia [6]. Body weight represents one’s size and physical characteristics; it constitutes an external part of body image. Body weight also forms an essential impression upon initial contact with others. Because of the influence of peers and media figures, most female college students imbibe “thinness” as an ideal image [7,8,9]. They possess a heightened body weight awareness [8,10,11]. Female college students are less satisfied with their appearance, even when their Body Mass Index (BMI) is within the normal range [9].

Insufficient exercise may cause overweight and obesity [12]. College students spend most of their time using computers [5] and smartphones [13]. College students use these devices to fulfill course requirements; they also use streaming and social networking platforms, such as Netflix, YouTube, and Instagram, to relax or to distract them from stress and anxious or depressive moods [14,15].

Stress and negative moods may further affect diet and exercise behaviors. People possess distinct personality traits and react differently to stress and negative moods [16]. People usually celebrate good or happy events with an abundant meal. Under stress or negative moods, some people may manage it by eating more or less [17,18] or participating more or less in exercise [19,20]. 

Meanwhile, higher perceived stress increases vulnerability to mental disturbance, such as anxiety, depression, and poor sleep [20,21]. College students perceive higher stress than the normative group [22,23]. Their mean scores on the Perceived Stress Scale (PSS) range from 16.3 to 20.9 [14,22,24,25]. As per studies using various screening tools, the percentage of college students demonstrating mental disturbances ranges from approximately 49.6 to 56.6% [25,26]. In nursing education, students identify academic load and clinical practicums as stressors in Scotland [27], Japan [28], China [29], and Turkey [26]. Prior to clinical practicums, students must achieve the requirements of laboratory (nursing skills) courses. However, stressors may vary in different cultural contexts. For example, finance is an identified stressor in Scotland [27] and China [29] but not in Japan [28]. 

Nursing is a helping profession. Because of few breaks and rotating shifts, nurses have increased risks of overweight and obesity [30,31]. In becoming a nurse, students must also learn to care for themselves. They will have a better opportunity to deal with clinical demands in the future [30]. Few studies have investigated nursing college students’ body weight. In this population, studies regarding the influences of BMI, body image, perceived stress, and mental health as a whole are still lacking. The study findings will help nurse educators re-examine the course designs and the necessity of course adjustment. An intervention plan will be initiated to promote nursing college students’ health and well-being. Based on the research findings mentioned above, we hypothesized that (a) there will be associations among BMI, body image, perceived stress, mental health, dietary habits, and exercise participation; (b) there will be differences in body image, perceived stress, mental health, dietary habits, and exercise participation among BMI subgroups. Therefore, the preliminary study aimed to (a) investigate the associations of BMI, body image, perceived stress, mental health, dietary habits, and exercise participation; (b) examine the differences in body image, perceived stress, mental health, dietary habits, and exercise participation among BMI subgroups.

## 2. Materials and Methods

### 2.1. Design

The study used a cross-sectional design with convenience sampling.

### 2.2. Setting and Sample

Considering the follow-up intervening access, this study recruited female nursing students from a university located in a rural area of southern Taiwan. From previous studies, we know that:

- Finance [27,29] and practicum [26,27,28,29] may increase students’ stress levels;
- Older or married students may take more responsibility for caring for their families [20,21];
- First-year students may perceive more stress [32].

At this university, most full-time students from the day section received financial support from their families, while some received loans from the Ministry of Education, Taiwan. The students in the continuing education division usually had full- or part-time jobs; meanwhile, some were married and took more responsibility for caring for their families. Moreover, the Civil Code in Taiwan used to regulate that people aged at least 20 years could sign informed consent independently (this regulation changed to age 18 years on 1 January 2023).

To minimize the above confounding factors, we set up the inclusion criteria as follows: (a) aged 20–25 years, (b) studying full-time in a two-year or four-year bachelor’s degree program in the day section, and (c) studying at school without clinical practicum. The exclusion criteria were (a) first-year students in a four-year bachelor’s degree program and (b) students in the continuing education division. The G*Power Version 3.1.9.7 estimated the sample size [33]. The minimum sample size was 191, determined by correlation analysis with two tails, an effect size of 0.2, α of 0.05, and a power of 0.8.

### 2.3. Data Collection and Measures

#### 2.3.1. Procedure

The study protocol was approved by the Institutional Review Board of the Medical Foundation (IRB No.: 201701084B0) and then by the Research and Development Division at the university in Taiwan. We then approached students in class meetings after obtaining permission from their mentors. Before explaining the study, students were informed that their participation was voluntary and would not influence their academic scores. Meanwhile, students were told they would be free to leave if they were uninterested. If they were in the researchers’ class, other researchers, who did not participate in this study, would explain it.

We explained to students the nature of the study, e.g., voluntary participation, no added or deducted academic score for participation, study purpose, questionnaires, possible risks, withdrawal from the study at any time, informed consent, confidentiality, and anonymity. Pairing codes printed on the informed consent and the questionnaires kept the participants’ confidentiality and were analyzed anonymously. All participants filled out the questionnaires after understanding and signing the informed consent. Each participant received an incentive reward worth approximately CHF 1.5 (Swiss Francs) for filling out the questionnaires. Self-reported paper–pencil questionnaires were collected from October 2017 to May 2018.

#### 2.3.2. Demographic Data

Demographic data included age, bachelor’s degree program, accommodation, computer and smartphone use, weight management experience, alcohol and cigarette use, dietary habits, exercise participation, and self-reported and expected body height (cm) and body weight (kg). 

Dietary habits assessed the frequency of food choices (four items) and eating situations (four items), modified based on Ho et al. [34]. Two examples of food choices items were “How often do you avoid eating fried food, e.g., fried chicken chop, French fried, fried chicken, and Taiwanese popcorn chicken?” and “How often do you avoid eating processed meat, e.g., sausage, ham, and bacon?” Two examples of eating situations items were “How often do you eat under stress or negative moods?” and “How often do you have snack attacks while not being hungry?”. The frequency was rated from 1 (rarely) to 4 (always). The score ranged from 8 to 32, with a higher score indicating better dietary habits.

Exercise participation was grossly estimated by the formulation of frequency (per week)×(duration+intensity), adapted from Yeh and Chang [35]. Yeh and Chang [35] simplified and modified this formulation based on the study of Fox [36]. The duration was categorized into ≤30 (scoring 1), 31–60 (scoring 2), 61–90 (scoring 3), 91–120 (scoring 4), and >120 min (scoring 5). The intensity was assessed by how tired respondents felt after exercise, from 1 (not tired at all) to 5 (very tired). A higher score represented better exercise involvement. Respondents were also asked whether they exercised alone.

#### 2.3.3. Body Mass Index

The formula of BMI (kg/m^2^) is body weight (kg) ÷ [body height (m)×body height (m)]. This study classified four BMI subgroups based on the cut-offs suggested by the Regional Office for the Western Pacific of the World Health Organization [37] (Table 1).

#### 2.3.4. Multidimensional Body-Self Relations Questionnaire—Appearance Scale

Body image was appraised by a 34-item appearance scale of the Multidimensional Body–Self Relations Questionnaire (MBSRQ-AS) [38]. The MBSRQ-AS contains Appearance Evaluation (AE) (7 items), Appearance Orientation (AO) (12 items), Body Area Satisfaction (BAS) (9 items), Overweight Preoccupation (OP) (4 items), and Self-Classified Weight (SCW) (2 items). A response scale from 1 (definitely disagree) to 5 (definitely agree) was used to assess the levels of agreement or satisfaction, except for items 23–25. Item 23 evaluates the frequency of diet control to reduce body weight, from 1 (never) to 5 (very often). Respondents self-evaluated their body weight on items 24–25, from 1 (very underweight) to 5 (very overweight). A higher mean score on each subscale represents respondents’ satisfaction, value, and concern toward the specific subdomain, except for SCW. The SCW assesses how respondents evaluated their body weight and perceived “most” other people’s viewpoints. A higher mean score on SCW indicates that respondents’ body weight was categorized as heavier. The original Cronbach’s α for each subscale was 0.80, 0.83, 0.79, 0.70, and 0.84, respectively [38]. The Cronbach’s α for each subscale in this study was 0.80, 0.83, 0.78, 0.70, and 0.83, respectively.

#### 2.3.5. Perceived Stress Scale

The stress level was measured by the 10-item PSS [23]. Compared with the 4- and 14-item PSS, the 10-item version reflects better on positive and negative perceptions [39]. A response scale from 0 (never) to 4 (very often) was used to assess the frequency of these perceptions. Its score ranges from 0 to 40; a higher score indicates a higher stress level. The normative datum for women was 13.7. The original Cronbach’s α was 0.78 [23]; the Cronbach’s α in this study was 0.86. 

#### 2.3.6. Chinese Health Questionnaire

Mental health was measured by a 12-item Chinese Health Questionnaire (CHQ) [40]. The CHQ aims to screen mental disturbance in the Taiwanese community. Therefore, it reflects Taiwanese culture in two approaches: family-centered and somatic complaints. CHQ assesses somatic symptoms, anxiety/worry, depression, poor relations with family or close friends, and sleep [40,41]. It measures frequency compared to daily life, from 1 (not at all), 2 (same as usual), 3 (rather more than usual), to 4 (much more than usual). The original score ranges from 12 to 48; a higher score represents a poorer mental health status. 

Because the frequency compares to usual daily life, it finally scores “0” for “not at all” and “same as usual”, and “1” for “rather more than usual” and “much more than usual”, to detect community people suffering from mental disturbance. The transforming score ranges from 0 to 12; the cut-point score for mental disturbance is equal to or greater than 3 [40]. The original Cronbach’s α was 0.84 in the community population [41]; the Cronbach’s α in this study was 0.76.

### 2.4. Data Analysis

Data were analyzed by IBM SPSS Statistics (version 28). A total of 310 female nursing students completed the questionnaires; 40 participants were excluded because of inappropriate responses (filling out the same answers; *n* = 6) and missing data (*n* = 34). The following statistical methods were performed by the complete case analysis (i.e., listwise deletion) to exclude missing data [42]. Cronbach’s alpha was used to measure the internal consistency of MBSRQ-AS, PSS, and CHQ. Categorical variables were presented as frequencies and percentages. Continuous variables did not pass the Kolmogorov–Smirnov test for normality and were presented as the median and interquartile range. The non-parametric methods were then performed [43]. Spearman correlation was performed to identify associations among self-reported BMI and other variables. The Wilcoxon signed-rank test was employed to examine the difference between self-reported and expected BMI. The Kruskal–Wallis test was used to detect the differences among BMI subgroups; post hoc analyses were then examined.

## 3. Results

### 3.1. Characteristics of the Participants

The median age of the 310 participants was 21 years. They mainly lived in the dormitory (98.4%) (Table 2). Only 4.5% consumed alcohol and 0.3% had smoked in the previous month upon filling out the questionnaires. They were prone to choose food and drinks with high fat and carbohydrate/sugar levels, eat for rewarding self or celebration, and enjoy delicious food in front of them. However, 62.6% reported they rarely or sometimes relieved negative moods or stress by eating. A total of 61.9% reported they rarely or sometimes satisfied their snack attacks by eating when not hungry. Time spent in computer and smartphone-related activities was dramatically individualized, particularly for smartphone use.

Furthermore, 27.4% of the participants did not exercise. Although 72.6% exercised on various levels and often with companions, 29.8% participated in exercise less than the median of 10. In other words, almost half of the participants needed to exercise more. 

**Figure 1:**
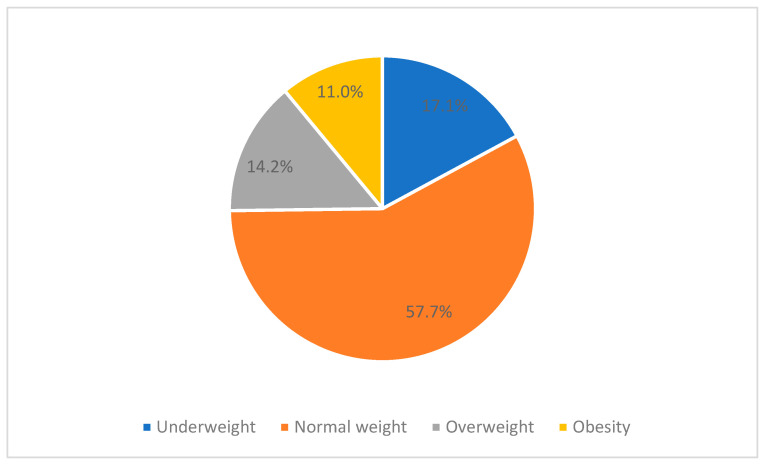
The prevalence rates of underweight, normal weight, overweight, and obesity.

This study categorized BMI subgroups based on the suggestions of the Regional Office for the Western Pacific of the World Health Organization [37] (Table 1). The prevalence rates of underweight, overweight, and obesity were 17.1%, 14.2%, and 11.0%, respectively, while the rate of normal weight was 57.7% (Figure 1). Most participants (81.9%) had weight management experience. Among 56 participants (18.1%) without weight management experience, the number of underweight, normal weight, overweight, and obesity were 31, 19, 2, and 4, respectively. The median self-reported BMI was 20.83 kg/m^2^; the median expected BMI was 18.37 kg/m^2^. Wilcoxon signed-rank test examined the difference between self-reported and expected BMI. The difference was significant (Z = −14.21, *p* < 0.001); the participants hoped to have a lower BMI.

The participants tended to be appearance-oriented. However, they were prone to be neutral regarding their appearance and body area (e.g., upper, mid, and lower torso, face, and hair). Although they classified their body weight in the middle of “normal weight” and “somewhat overweight”, their attitude concerning being overweight was prone to be neutral.

The participants tended to feel stressed sometimes. They tended to experience depressive moods, poor relations with family or close friends, and poor sleep. Therefore, 45.2% experienced “mental disturbance” based on the Chinese Health Questionnaires cut-point score [40].

### 3.2. Associations of Self-Reported BMI and Other Variables

Table 3 shows the results of the Spearman correlation. Self-reported BMI was unrelated to computer and smartphone use, exercise participation, dietary habits, mental health, and perceived stress. However, the higher the self-reported BMI was, the lower the satisfactory scores were on appearance (*r_s_* = −0.33, *p* < 0.01) and body area (e.g., upper, mid, and lower torso, face, and hair) (*r_s_* = −0.34, *p* < 0.01). The higher the self-reported BMI was, the higher the concern score was on overweight preoccupation (*r_s_* = 0.38, *p* < 0.01), the heavier the self-classified weight was (*r_s_* = 0.80, *p* < 0.01), and the higher the expected BMI was (*r_s_* = 0.71, *p* < 0.01).

There was a meaningful association between mental health and perceived stress (*r_s_* = 0.55, *p* < 0.01). When mental health was poor and perceived stress was high, the satisfactory scores on appearance and body area became low. The higher the perceived stress was, the higher the concern score on overweight preoccupation (*r_s_* = 0.20, *p* < 0.01), and the heavier the self-classified weight was (*r_s_* = 0.15, *p* = 0.009).

### 3.3. Differences in Variables among BMI Subgroups

Table 4 presents the results of the Kruskal–Wallis test. There were differences in appearance evaluation, body area satisfaction, overweight preoccupation, self-classified weight, and expected BMI among the underweight, normal weight, overweight, and obesity groups. The normal weight group was more satisfied with their appearance and body area (e.g., upper, mid, and lower torso, face, and hair) than the obesity group. However, the underweight group had the highest satisfactory scores (*p* < 0.001). There were no significant differences in overweight preoccupation among the groups of normal weight, overweight, and obesity, whereas the underweight group had the lowest concern regarding being overweight (*p* < 0.001). There were no meaningful differences between the groups of overweight and obesity regarding body image. The underweight group expected the lowest BMI; this group also classified their body weight as the lowest, followed by the normal weight group (*p* < 0.001). In short, the underweight group had the highest satisfactory score regarding body image.

## 4. Discussion

The present study found that:

- Self-reported BMI had no significant associations with computer and smartphone use, exercise participation, dietary habits, mental health, and perceived stress;
- Self-reported BMI was correlated to body image and expected BMI;
- There were differences in body image and expected BMI among BMI subgroups.

### 4.1. Associations of Self-Reported BMI and Other Variables

We were surprised that the correlational analysis results differed markedly from the conclusions in the literature [3,4,5,14], except for body image. Computer and smartphone use increases inactivity and decreases exercise participation, particularly under negative moods [5,13,14]. However, studies [3,4,17] indicated that dietary habits influence BMI or body weight more than exercise participation. Commonly, college students choose food and drinks with high fat and carbohydrate/sugar levels because of personal preference and time-saving [3,4]. Furthermore, most students staying at the dormitory rely on take-out services and campus-based cafeterias [3].

However, whether people react to stress and negative moods by eating may be an external factor causing increased body weight or BMI [17]. Haidar et al. [18] reported inconsistent evidence regarding the relationship between stress or negative moods and weight gain. Pfeiler and Egloff [16] noted that people with distinct personalities behave differently in eating. They found people characterized with an “emotional stability” personality presented a lower BMI.

Eating behaviors in response to stress and negative moods may contribute to the discrepancy in study findings. The participants we recruited tended to choose not to eat when not hungry and in negative moods. However, Yönder Ertem and Karakaş [44] found that female participants were likely to eat when not hungry and in negative moods. The behavior was related to a lack of self-confidence in coping [44]. Choi [22] also reported a similar finding in female students’ eating behaviors.

Students’ academic year and study majors may influence their eating behaviors or coping strategies. The participants we recruited comprised 43.8% at least third-year students in the four-year bachelor’s degree program and 53.2% in the two-year bachelor’s degree program. The latter had completed a five-year associate bachelor’s degree. Previous studies [22,44] recruited various academic year students. Senior nursing students might know more about coping with stress and negative moods. Jiang et al. [3] also revealed that health-related major’s students had more knowledge regarding healthy behaviors and practiced better than non-health-related major’s students. Of course, the present study used convenience sampling on a small scale and was conducted in one university, which may cause discrepancies from other studies.

Similar to previous studies [20,21,25], the higher the stress the female nursing college students perceived, the poorer their mental health. The present study also found that their perceived stress was higher [21,25] than the normative group [23]. We found the prevalence of mental disturbance, e.g., anxiety, depression, and poor sleep was 45.2% based on the Chinese Health Questionnaire. Despite different screening tools, Zhang et al. [25] revealed that 54.5% of 242 nursing college students suffered from depressive moods, while 56.6% reported having anxiety symptoms in the northern United States. We also found that the students were likely to evaluate their body image negatively under high perceived stress levels and negative moods. The high perceived stress and mental disturbance in nursing college students warrant attention.

Similarly, the present study found a close relationship between body image and BMI [8,9]. Female college students are likely to be dissatisfied with their appearance [7,8,9] and BMI or body weight [8,44] and being underweight [3,5]. College students are in young adulthood; their main task is to build close relationships through knowing others and developing friendships and love [45]. In addition to the influence of media and peer pressure [7,9], these forces push them to pursue a “thinness” image. We found that the underweight prevalence was 17.1%. Similarly, Jiang et al. [3] reported that the underweight prevalence was 24.6% among 692 female college students in Beijing, China. Yönder Ertem and Karakaş [44] reported that the underweight rate was 11.4% among 389 female and 84 male nursing college students in Turkey. Although being underweight causes fewer health problems than being overweight and obese [2], its prevalence among female college students in some study populations should be noted with caution.

### 4.2. Differences in Variables among BMI Subgroups

The significant correlation between body and image was also demonstrated in the differences among BMI subgroups. Undoubtedly, the underweight group had the highest satisfaction with their appearance and body area (e.g., upper, mid, and lower torso, face, and hair). We had known that the participants expected to have a higher body height and a lower body weight, which resulted in a lower expected BMI. The phenomenon also explained that weight management experience was common among our participants. 

It was interesting to find no significant differences in body image between the overweight and obese groups. Other than what we discussed in the section on “associations among BMI and other variables”, we did not examine or follow the changes in students’ eating and exercise behaviors from their first year in the nursing educational program. The longitudinal study might provide a better understanding of the influence of health-related education among nursing students. 

**Figure 2:**
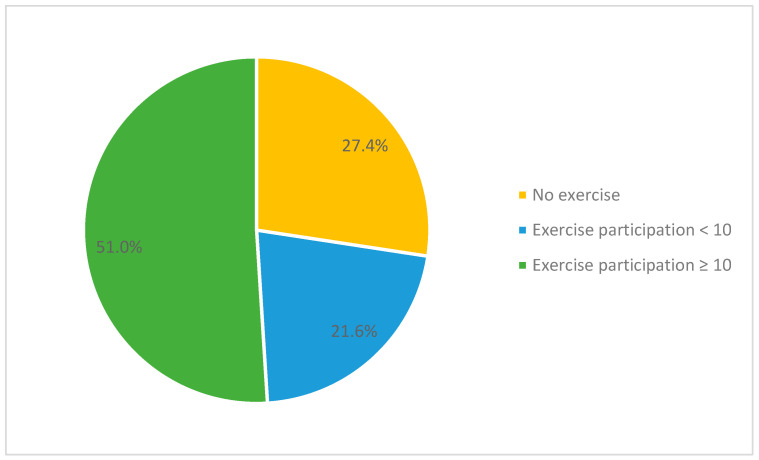
Categorized excise participation into three groups: (1) No exercise, (2) Exercise participation < 10, and (3) Exercise participation ≥ 10.

Exercise participation was insignificantly different among BMI subgroups. However, compared with the median of 10 in exercise participation among 310 participants (Table 2), the underweight and obese group needed more exercise. Considering its interquartile ranges among all BMI subgroups (Table 4), we further categorized the participants’ scores on exercise participation (Figure 2); 49% of the participants scored 0 to 9 in exercise participation. Although exercise might not play an essential role in causing increased BMI or body weight among young college students [3,17], it helps individuals maintain or improve fasting blood sugar, blood pressure, and cholesterols within normal limits, even though they are obese [46]. 

### 4.3. Suggestions and Limitations

From this preliminary study, the interventions target (1) body image, (2) dietary habits, (3) perceived stress and mental health, and (4) exercise through individual and group approaches.

#### 4.3.1. Body Image

Body image is built upon one’s and others’ evaluations regarding oneself [38]. It is essential to discuss body image, beauty, body weight management, and health outcomes related to underweight, overweight, and obesity in group approaches. Before group discussion, students should calculate their actual and ideal body weight into BMI. Measuring body weight is more accessible than obtaining BMI. People usually overlook BMI values because such data are acquired after keying body height into the measuring tool or being measured by an automatic height–weight BMI machine. Students should recognize and appreciate that their body parts endow them freely with various functions and abilities [47]. 

#### 4.3.2. Dietary Habits

A balanced diet is essential to ensure that students intake adequate nutrients. Education institutes should regularly monitor the contents and nutrients of food and drinks at campus-based cafeterias [4]. Nursing students have learned to educate patients with diabetes on calculating calories, substituting one type or proportion of food with another, and gradually increasing physical activities. They could use similar strategies to decrease or limit the frequency or amount of food and drinks with high fat and carbohydrate/sugar levels.

#### 4.3.3. Perceived Stress and Mental Health

It is necessary to incorporate coping strategies into the curriculum [26]. Students should learn to cope with stress and negative moods more healthily than by eating. Furthermore, assessing nursing students’ psychological status might be necessary before clinical practicums, particularly those with difficulty succeeding in laboratory courses. With proper mental and learning support from peers, faculties, and education institutes [20,21,26], their stress and negative moods might mitigate. They then may sleep better [25] and they might have more opportunities to pass through stress and negative moods.

#### 4.3.4. Exercise

Sufficient exercise is an excellent buffer for maintaining and promoting physical and mental health [19,46]. Exercise requires time, as does completing school-related assignments and examinations [48]. Time management is essential for incorporating exercise into students’ schedule planning [48]. 

Meanwhile, faculties should encourage students:

- To walk around or stretch muscles instead of napping on tables or sitting in their seats during class breaks [49];
- To utilize at least one flight of stairs instead of escalators or elevators [50].
- To commute by public transportation as much as possible [51];
- To increase their walking or use of school- or community-based fitness facilities with peers or family through smartphone apps and social support [48,52].

Education institutes and local governments could promote physical activities by posting motivational signs to encourage the use of staircases [53] and by holding school- or institute-based games/sports/running/dance competitions [54].

#### 4.3.5. Limitations

This study has some limitations. First, convenience sampling may cause selection bias, although the study sample size represented 44.7% of approximately 700 students aged equal to or greater than 20 who were enrolled in the university’s bachelor program day section. Recruiting participants from only a university’s academic department further decreased the findings’ generalizability. This may contribute to the non-normal distribution data in our data. The interpretation of this preliminary study’s findings should be taken with caution. Second, measuring the participants’ height and body weight would prevent them from over- or under-estimating reports on their anthropometric data. Third, the Chinese Health Questionnaire is culturally sensitized to detect mental disturbance among Taiwanese populations. However, this might limit comparisons among nursing students from different countries or cultures.

Finally, a cross-sectional design might limit understanding regarding the associations among BMI, body image, perceived stress, mental health, dietary habits, and exercise participation. We only used four items (“eating situations”) to assess eating behaviors under certain situations. Moreover, we did not know the actual causal relationship among these factors. The inconsistent study findings illuminate the complexities of appropriate weight management. A longitudinal study may consider incorporating coping strategies or personality into the study design and conducting on a large scale by a stratified sampling on academic years, health-related and non-health-related majors. The findings will benefit planning health interventions and course design.

## 5. Conclusions

The preliminary study found that female nursing college students hoped to have a lower BMI. A total of 42.3% of them were classified as underweight, overweight, and obese. The students with higher perceived stress might experience mental disturbance. Therefore, faculties and education institutes should focus more on students’ body image, coping strategies, time management, balanced diets, and sufficient exercise. As nursing students develop better abilities and capacities to “juggle” daily activities [21], their experiences would potentially benefit them during clinical practicums [55]. These strategies and practices might further equip them to balance their personal and professional lives [21].

## Figures and Tables

**Table 1:** BMI classifications.

Weight Status	World Health Organization [2]	The Regional Office for the Western Pacific of the World Health Organization [37]
Underweight	BMI < 18.5 kg/m^2^	BMI < 18.5 kg/m^2^
Normal weight	18.5 kg/m^2^ ≤ BMI < 25 kg/m^2^	18.5 kg/m^2^ ≤ BMI < 23 kg/m^2^
Overweight	25 kg/m^2^ ≤ BMI < 30 kg/m^2^	23 kg/m^2^ ≤ BMI < 25 kg/m^2^
Obesity	BMI ≥ 30 kg/m^2^	BMI ≥ 25 kg/m^2^

Note. BMI—Body Mass Index.

**Table 2:** Characteristics of the participants (N = 310).

Variable	*n*	%	Median	IQR
** Bachelor’s degree program **				
(1) Four-year program	145	46.8		
Second-year students	9	2.9		
Third-year students	94	30.3		
Fourth-year students	42	13.5		
(2) Two-year program ^†^	165	53.2		
First-year students	131	42.3		
Second-year students	34	11.0		
** Accommodation **				
Dormitory	305	98.4		
Rented room	2	0.6		
Home (with parents)	3	1.0		
** Alcohol use in previous month **				
No	296	95.5		
Yes	14	4.5		
** Cigarette use in previous month **				
No	309	99.7		
Yes	1	0.3		
** Weight management experience **				
No	56	18.1		
Yes	254	81.9		
** Purpose of weight management **				
Increase weight	2	0.6		
Decrease weight	201	64.8		
Maintain weight	51	16.5		
Non-applicable	56	18.1		
** Weight management strategies (multiple response type) **				
Diet control	223	71.9		
Exercise control	213	68.7		
Western medication	10	3.2		
Auricular acupressure	2	0.6		
** Exercise with a companion **				
Yes	175	56.5		
No	50	16.1		
Non-applicable (no exercise)	85	27.4		
** Exercise participation **			10	15
** Computer use (h/wk) **			10	16
** Smartphone use (h/wk) **			28	35
** Dietary habits **			19	6
Food choices			9	4
Eating situations			10	3
** Self-reported body height (cm) **			160	8
** Expected body height (cm) **			163	5
** Self-reported body weight (kg) **			54	10
** Expected body weight (kg) **			50	6
** Self-reported BMI (kg/m^2^) **			20.83	3.66
** Expected BMI (kg/m^2^) **			18.37	1.90
** PSS **			18	7
** MBSRQ-AS **				
Appearance evaluation			3.14	0.71
Appearance orientation			3.67	0.67
Body area satisfaction			3.00	0.67
Overweight preoccupation			2.75	1.00
Self-classified weight			3.50	1.00
** CHQ (original scores) **			21	7
Somatic symptoms			5	3
Anxiety/worry			4	2
Depression and poor relations			10	3
Poor sleep			2	1
** CHQ (transforming scores) **			2	1
Somatic symptoms			0	0
Anxiety/worry			0	1
Depression and poor relations			2	1
Poor sleep			2	1
Normal status: ≤2	170	54.8		
Mental disturbance: ≥3	140	45.2		

Note. IQR—Interquartile Range; BMI—Body Mass Index; PSS—Perceived Stress Scale; MBSRQ-AS—Multidimensional Body–Self Relations Questionnaire—Appearance Scale; CHQ—Chinese Health Questionnaire (mental health). ^†^ The two-year bachelor’s degree program accepts students from a five-year associate bachelor’s degree program in nursing.

**Table 3:** Spearman correlational matrix between self-reported BMI and other variables.

	Variable	1	2	3	4	5	6	7	8	9	10	11	12
1	Self-reported BMI	–											
2	Computer use	0.00	–										
3	Smartphone use	−0.11	0.28 **	–									
4	Exercise participation	0.04	0.01	−0.06	–								
5	Dietary habits	−0.04	0.00	−0.17 **	0.05	–							
6	CHQ	0.00	0.11	0.02	−0.05	−0.07	–						
7	PSS	0.04	0.09	0.03	−0.02	−0.20 **	0.55 **	–					
8	AE	−0.33 **	−0.09	0.03	0.01	0.14 *	−0.24 **	−0.24 **	–				
9	AO	−0.02	−0.01	0.18 **	0.08	−0.09	−0.04	−0.02	0.20 **	–			
10	BAS	−0.34 **	−0.10	−0.03	0.07	0.18 **	−0.23 **	−0.26 **	0.66 **	−0.03	–		
11	OP	0.38 **	−0.06	−0.08	0.10	−0.08	0.09	0.20 **	−0.15 **	0.36 **	−0.32 **	–	
12	SCW	0.80 **	0.08	−0.07	0.08	−0.06	0.08	0.15 **	−0.40 **	0.04	−0.46 **	0.44 **	–
13	Expected BMI	0.71 **	0.02	−0.10	0.05	0.05	−0.05	−0.06	−0.11 *	−0.09	−0.07	0.12 *	0.52 **

Note. BMI—Body Mass Index; CHQ—Chinese Health Questionnaire (mental health); PSS—Perceived Stress Scale; AE—Appearance Evaluation; AO—Appearance Orientation; BAS—Body Area Satisfaction; OP—Overweight Preoccupation; SCW—Self-Classified Weight. * p < 0.05, two-tailed. ** *p* < 0.01, two-tailed.

**Table 4:** The differences among BMI subgroups by the Kruskal–Wallis test (N = 310).

	Underweight ^a^ (n = 53)		Normal weight ^b^ (n = 179)		Overweight ^c^ (n = 44)		Obesity ^d^ (n = 34)				
Variable	Median	(IQR)	Median	(IQR)	Median	(IQR)	Median	(IQR)	*p*	Post Hoc	
** Computer use **	8	(20)	12	(16)	9.5	(21)	14	(15)	0.73		
** Smartphone use **	30	(38)	28	(34)	26	(23.25)	28	(45.75)	0.26		
** Exercise participation **	8	(13.25)	10	(15)	10	(15)	9	(17)	0.84		
** Dietary habits **	19	(6)	19	(6)	19.5	(6.75)	18	(6.5)	0.59		
** PSS **	17	(6.5)	18	(7)	19	(5.75)	16.5	(6)	0.62		
** CHQ **	21	(8.5)	21	(5)	20	(6.75)	21	(8)	0.94		
** MBSRQ-AS **											
AE	3.29	(0.57)	3.14	(0.86)	2.86	(0.68)	2.64	(0.86)	<0.001	a > b, c, d; b > d	
AO	3.67	(0.83)	3.67	(0.67)	3.67	(0.48)	3.58	(0.69)	0.92		
BAS	3.33	(0.83)	3.00	(0.67)	2.78	(0.67)	2.78	(0.72)	<0.001	a > b, c, d; b > d	
OP	2.00	(1.13)	2.75	(0.75)	3.13	(0.75)	3.25	(0.75)	<0.001	a < b, c, d	
SCW	2.00	(1.25)	3.50	(0.50)	4.00	(0.38)	4.50	(1.00)	<0.001	a < b < c, d	
** Expected BMI **	17.58	(1.36)	18.37	(1.38)	20.30	(1.76)	21.48	(3.09)	<0.001	a < b < c, d	

Note. IQR—Interquartile Range; BMI—Body Mass Index; PSS—Perceived Stress Scale; CHQ—Chinese Health Questionnaire (mental health); MBSRQ-AS—Multidimensional Body–Self Relations Questionnaire—Appearance Scale; AE—Appearance Evaluation; AO—Appearance Orientation; BAS—Body Area Satisfaction; OP—Overweight Preoccupation; SCW—Self-Classified Weight. ^a^ Underweight = BMI < 18.5 kg/m^2^. ^b^ Normal weight = 18.5 kg/m^2^ ≤ BMI < 23 kg/m^2^. ^c^ Overweight = 23 kg/m^2^ ≤ BMI < 25 kg/m^2^. ^d^ Obesity = BMI ≥ 25 kg/m^2^.

## Data Availability

Data available on request from the authors.
